# Exendin-4 Improves Steatohepatitis by Increasing Sirt1 Expression in High-Fat Diet-Induced Obese C57BL/6J Mice

**DOI:** 10.1371/journal.pone.0031394

**Published:** 2012-02-17

**Authors:** Jinmi Lee, Seok-Woo Hong, Seoung Wan Chae, Dong Hoon Kim, Ji Hun Choi, Ji Cheol Bae, Se Eun Park, Eun-Jung Rhee, Cheol-Young Park, Ki-Won Oh, Sung-Woo Park, Sun-Woo Kim, Won-Young Lee

**Affiliations:** 1 Institute of Medical Research, Kangbuk Samsung Hospital, Sungkyunkwan University School of Medicine, Seoul, Korea; 2 Department of Pathology, Kangbuk Samsung Hospital, Sungkyunkwan University School of Medicine, Seoul, Korea; 3 Department of Endocrinology and Metabolism, Kangbuk Samsung Hospital, Sungkyunkwan University School of Medicine, Seoul, Korea; Paris Institute of Technology for Life, Food and Environmental Sciences, France

## Abstract

The effects of exendin-4 on Sirt1 expression as a mechanism of reducing fatty liver have not been previously reported. Therefore, we investigated whether the beneficial effects of exendin-4 treatment on fatty liver are mediated via Sirt1 in high-fat (HF) diet-induced obese C57BL/6J mice and related cell culture models. Exendin-4 treatment decreased body weight, serum free fatty acid (FA), and triglyceride levels in HF-induced obese C57BL/6J mice. Histological analysis showed that exendin-4 reversed HF-induced hepatic accumulation of lipids and inflammation. Exendin-4 treatment increased mRNA and protein expression of Sirt1 and its downstream factor, AMPK, *in vivo* and also induced genes associated with FA oxidation and glucose metabolism. In addition, a significant increase in the hepatic expression of *Lkb1* and *Nampt* mRNA was observed in exendin-4-treated groups. We also observed increased expression of phospho-Foxo1 and GLUT2, which are involved in hepatic glucose metabolism. In HepG2 and Huh7 cells, mRNA and protein expressions of GLP-1R were increased by exendin-4 treatment in a dose-dependent manner. Exendin-4 enhanced protein expression of Sirt1 and phospho-AMPKα in HepG2 cells treated with 0.4 mM palmitic acid. We also found that Sirt1 was an upstream regulator of AMPK in hepatocytes. A novel finding of this study was the observation that expression of GLP-1R is proportional to exendin-4 concentration and exendin-4 could attenuate fatty liver through activation of Sirt1.

## Introduction

Insulin resistance is an important mechanism underlying type 2 diabetes mellitus (T2DM), and recently, non-alcoholic fatty liver disease (NAFLD) has been reported to be associated with metabolic diseases such as T2DM, obesity, hypertension, and insulin resistance [1]. In clinical studies, it has been shown that weight loss can improve fatty liver, and that reduced liver fat content confers lower serum fasting insulin and triglyceride (TG) concentrations compared to subjects with high levels of liver fat [2]. Thus, fat accumulation in the liver is an important factor for the development of insulin resistance and dyslipidemia.

Glucagon-like peptide (GLP)-1, an incretin secreted by L-cells in the small intestine in response to food intake, is known to improve insulin secretion and its effects on reduction of appetite and body weight have been demonstrated in both rat [3] and human studies [4]. Thus, the administration of GLP-1 has been proposed as a therapeutic approach for T2DM. However, the half-life of exogenously administered bioactive GLP-1 is less than 2 minutes in rodents and humans due to its rapid inactivation by circulating dipeptidyl peptidase-IV (DPP-IV) [5].

Exenatide (exendin-4, Ex-4), a GLP-1 receptor (GLP-1R) agonist, shares 53% sequence homology with native GLP-1. Exendin-4 is resistant to DPP-IV mediated degradation, and therefore has a longer half-life than GLP-1 [6], [7]. Recent studies have shown that GLP-1R is present in human hepatocytes [8] and that administration of exendin-4 improves insulin resistance in *ob/ob* mice and reduces hepatic lipid storage [9]. In addition, exenatide therapy decreases fasting plasma glucose, body weight, and liver fat in patients with T2DM [10].

Silent mating type information regulation 2 homolog (sirtuin, SIRT) 1, one of the seven sirtuins identified in mammalian cells, is a NAD^+^-dependent histone/protein deacetylase that is activated in response to fasting and caloric restriction (CR). Resveratrol and SRT1720, both of which are Sirt1 activators, ameliorate fatty liver with reduced lipid synthesis and increased rates of fatty acid oxidation through Sirt1 and adenosine monophosphate-activated protein kinase (AMPK) activation [11]. In addition, activation of the Sirt1-forkhead box O1 (FOXO1) signaling pathway by resveratrol inhibits the expression of SREBP-1 in a cell model of steatosis induced by palmitate [12].

However, the effects of exendin-4 treatment on Sirt1 expression in a fatty liver model have not been previously reported. Therefore, we investigated whether the beneficial effects of exendin-4 treatment on fatty liver could be mediated via Sirt1 in high-fat (HF) diet-induced obese C57BL/6J mice and related cell culture models.

## Materials and Methods

### Animals

Six-week-old C57BL/6J mice were obtained from Central Laboratory (Shizuoka Laboratory Animal Center, Shizuoka, Japan) and bred under standard conditions with a 12-h light/dark cycle. All procedures were approved by the Ethics Committee for Animal Experiments of the Sungkyunkwan University Kangbuk Samsung Hospital (Approval ID: 201103022). Mice were randomly divided into 3 groups (n = 10/group) as follows: low-fat diet (control, 10 kcal % fat, 20 kcal % protein, and 70 kcal % carbohydrate); HF diet (HF, 45 kcal % fat, 20 kcal % protein, and 35 kcal % carbohydrate); and HF diet plus 1 nmol/kg/day exendin-4 (Sigma-Aldrich Corp., St. Louis, MO, USA) via intraperitoneal (IP) injection. For the former diet regimen, exendin-4 was injected every other day while saline was injected to the other groups every other day for 10 weeks. The mice were allowed access to their specific diet and water *ad libitum*. In all experiments, body weight and food intake were checked twice per week. In the 11^th^ week, after overnight fasting, the animals were sacrificed, and liver tissues were extracted, immediately frozen in liquid nitrogen, and stored at −80°C until RNA and protein extraction.

### Cell culture

HepG2 and Huh7 human hepatoma cell lines were purchased from American Type Culture Collection (ATCC, Manassas, VA, USA) and Korean Cell Line Bank (KCLB, Seoul, Korea), respectively, and cultured in 6-well plates in Dulbecco's modified Eagle's medium (DMEM) (Gibco, Grand Island, NY, USA) containing 10% fetal bovine serum (Gibco), 1% penicillin/streptomycin (Gibco) [8]. At 70–80% confluence, cells were pretreated with or without nicotinamide (10 mM) (Fluka AG, Buchs SG, Switzerland), an inhibitor of SIRT1, or Compound C (10 µM) (Calbiochem, La Jolla, Calif, CA, USA), an inhibitor of AMPK, in serum-free DMEM for 24 h. Afterwards, exendin-4 (50 or 100 nM) (Sigma-Aldrich Corp.) was added to the wells in 0.4 mM palmitic acid (Sigma-Aldrich Corp.), prepared as described previously [13].

### Oil red O staining

HepG2 and Huh7 cells were plated in chamber slides. Briefly, cells were washed 3 times with PBS, fixed for 30 minutes with 4% paraformaldehyde in PBS, stained with Oil-Red O for 1 hour, and then rinsed with distilled water. Finally, the cells were examined by light microscopy (magnification, ×400). For quantitative analysis of lipid accumulation, 1 mL isopropanol was added to the stained culture plate and the absorbance was recorded at 540 nm.

### Serum analysis

After overnight fasting, serum samples were obtained for analysis of free fatty acid (FFA) and triglyceride (TG). Serum samples were separated by centrifugation at 4°C and stored at −80°C until measurements were performed. Plasma concentrations were measured using an enzyme-based FFA quantification kit (BioVision, Mountain View, CA, USA) and a TG assay kit (Cayman Chemical, Ann Arbor, MI, USA).

### Total RNA extraction and real-time RT-PCR

Total RNA was extracted from liver tissue using Trizol reagent (Invitrogen, Carlsbad, CA, USA). Generation of cDNA was performed by reverse transcribing 2 µg of total RNA with moloney murine leukemia virus reverse transcriptase (MMLV-RT) and oligo (dT)12–18 primer (Invitrogen). Briefly, RNA was denatured for 10 minutes at 72°C, then immediately placed on ice for 5 minutes. Denatured RNA added to a mixture of MMLV-RT, MMLV-RT buffer, and dNTPs and incubated for 1 h at 42°C. Then, reagent was inactivated by heating at 95°C for 2 minutes. Synthesized cDNA was amplified by real-time PCR (Light-Cycler 480; Roche, Lewis, UK) using SYBR green (Invitrogen) and specific primers (Bioneer Co., Daejon, Korea). Each cycle consisted of denaturation at 94°C for 15 seconds, annealing at 55°C for 10 seconds, and extension at 72°C for 20 seconds. Quantification was performed using the comparative 2-(delta delta Ct) method, *i.e.*, expression levels for the target genes were normalized to the β-actin (*Actb*) of each sample.

### Western blot analysis

For western blot analysis, liver (100 mg wet weight) was homogenized at 4°C in 300 ml of RIPA buffer (Santa Cruz Biotechnology, Inc., Santa Cruz, CA, USA). After a 30 minute-incubation on ice, protein was obtained by centrifugation at 12,000 rpm for 20 minutes at 4°C. Extraction of nuclear proteins was performed using nuclear extraction kit (Cayman Chemical Co., Ann Arbor, MI). The samples containing 30 µg of protein were separated on 4%–12% bis-Tris Nupage gels (Invitrogen) and transferred to polyvinylidene difluoride membranes. After transfer, the membranes were incubated overnight at 4°C with antibodies against GLP-1R (H-55, sc-66911; Santa Cruz Biotechnology, Inc.), SIRT1 (H-300, sc-15404; Santa Cruz Biotechnology, Inc.), phospho-AMPKα (Thr172, #2531; Cell Signaling Technology, Danvers, MA, USA), phosphor-FOXO1 (pS^249^, 441245G; Invitrogen), glucose transporter 2 (GLUT2, H-67, sc-9117; Santa Cruz Biotechnology, Inc.), and β-actin (#4967; Cell Signaling Technology). Membranes were incubated with horseradish peroxidase conjugated secondary antibodies, and visualized using enhanced chemiluminescence western blotting detection reagents.

### Histological analysis

After anesthesia, liver tissues were removed from animals and fixed in 10% neutral buffered formalin and embedded in paraffin. Then, the paraffin blocks were cut on a microtome into 5 µm thick sections. After deparaffinization and dehydration, 5-µm thick sections were stained with hematoxylin (catalog no. GHS132; Sigma-Aldrich Corp.) and eosin (H&E, catalog no. HT110116; Sigma-Aldrich Corp.). Histological slides were examined by light microscopy (magnification, ×200). Histological features including steatosis, inflammation, and hepatocellular injury were histologically graded by two pathologists [14]. Hepatic triglycerides were extracted from frozen tissue and measured by enzymatic assays (Sigma). Values of TG were normalized to protein concentration.

### Statistical analysis

Data and results were reported as means ± SE. Statistical comparisons were performed with paired two-tailed *t*-tests. Values of P<0.05 were considered statistically significant unless otherwise indicated.

## Results

### Effects of exendin-4 treatment on body weight, food intake, and serum FFA and TG levels

**Figure 1:**
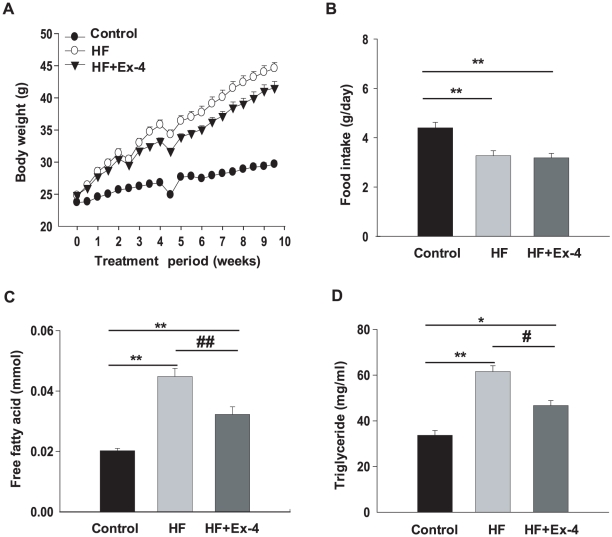
Change of body weight, food intake, and serum lipid levels by exendin-4 in HF-induced obese mice. Mice divided into 3 groups were fed low fat diet, high fat diet, or high fat diet with 1 nmol/kg/day exendin-4 ip injection for 10 weeks. (A) Body weight and (B) food intake was checked twice per week. (C–D) When sacrificed at 11 weeks, serum samples were obtained for analysis of free fatty acid (FFA) and triglyceride (TG) after overnight starvation. All values are expressed as the mean ± SE for n = 8 mice. * *p*<0.05, ** *p*<0.01 compared with a control and # *p*<0.05, ## *p*<0.01 compared with a HF.

The final body weight of the HF group was higher than that of the control group (45.2±0.72 g *vs.* 29.7±0.5 g in the control group) and that of the exendin-4-treated group (41±0.9 g) was significantly decreased compared with the HF group (Fig. 1A). However, despite the increase in body weight by HF, the food intake of HF group (3.27±0.19 g) was significantly decreased compared with the control group (4.4±0.22 g). There were no differences in food intake between the exendin-4-treated group (3.18±0.18 g) and the HF group (Fig. 1B). Exendin-4 treatment decreased the serum FFA level by 1.3-fold compared with the HF group (Fig. 1C). In addition, the TG level in the exendin-4-treated group was also decreased 1.3-fold compared with the HF group (Fig. 1D). The lowest levels of serum FFA and TG were noted in the control group.

### Exendin-4 treatment increases the hepatic expressions of GLP-1R, Nampt, Sirt1, Lkb1, and AMPKα

**Figure 2:**
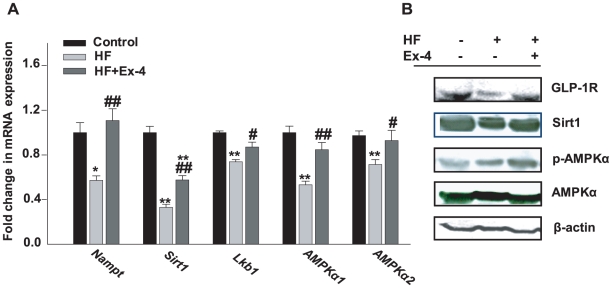
Change of Sirt1 and AMPK pathway and GLP-1R expression by exendin-4 in mouse livers. Total RNA and protein were extracted from liver tissue and (A) *Nampt*, *Sirt1*, *Lkb1*, *AMPK*α1 and *AMPK*α1 mRNA and (B) GLP-1R, Sirt1, phospho-AMPKα, AMPK and β-actin protein expression were measured by quantitative real-time RT-PCR and western blot, respectively. Data were normalized to the β-actin of each sample. All values are expressed as the mean ± SE for n = 5–7 mice. * *p*<0.05, ** *p*<0.01 compared with a control and # *p*<0.05, ## *p*<0.01 compared with a HF.

The effect of exendin-4 treatment, as a GLP-1R agonist, on the level of expression of GLP-1R was measured by western blot assay. Expression of GLP-1R in the HF group was decreased compared to that of the control group while exendin-4 treatment increased the expression of GLP-1R in liver tissue of mice compared with the HF group (Fig. 2B). In addition, the effect of exendin-4 treatment on mRNA and protein expression of hepatic Sirt1, Lkb1, and AMPKα was measured by real-time PCR and western blot assay. As shown in Fig. 2, mRNA expression of *Sirt1*, *Lkb1*, *AMPKα1, and AMPKα2*, as well as protein expression of Sirt1 and phospho-AMPKα, were significantly decreased in the HF group compared with the control group and were reversed by exendin-4 treatment. Recently, Revollo *et al.* [15] reported nicotinamide phosphoribosyltransferase (Nampt), functioning as a systemic NAD biosynthetic enzyme, regulates Sirt1 activity in mammalian cells. Therefore, we examined whether exendin-4 could affect the expression of Nampt in the liver. In the HF group, *Nampt* mRNA expression level was significantly decreased compared with the control group while exendin-4 treatment significantly increased the expression of *Nampt* mRNA compared with its expression in the HF group (Fig. 2A).

### Effect of exendin-4 treatment on the hepatic expressions of genes associated with fatty acid oxidation, lipogenesis, and glucose homeostasis

**Figure 3:**
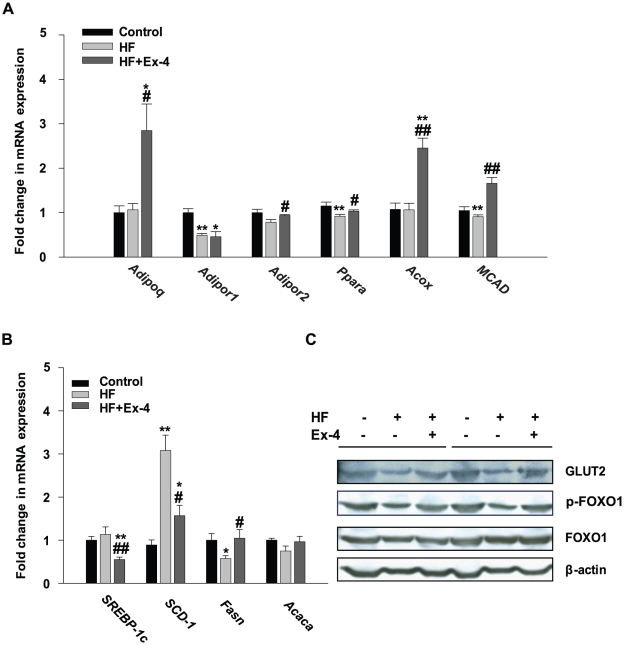
Effect of exendin-4 on expression of genes associated with fatty acid oxidation, lipogenesis, and glucose homeostasis. (A) Adiponectin (*Adipoq*), adiponectin receptor 1 (*AdipoR1*), adiponectin receptor 1 (*AdipoR2*), peroxisome proliferator-activated receptor- α (*Ppara*), acyl-CoA oxidase (*Acox*), *and* medium chain acyl-Coenzyme A dehydrogenase (*MCAD*); (B) sterol regulatory element binding protein 1c (*SREBP-1c*), stearoyl-Coenzyme A desaturase 1 (*Scd-1*), fatty acid synthase (*Fasn*), and acetyl-Coenzyme A carboxylase alpha (*Acaca*) mRNA expression; and (C) GLUT2, phosphorylated Foxo1 at serine 349, Foxo1 and β-actin protein expression in mouse livers were measured by quantitative real-time RT-PCR and western blot and were normalized to the β-actin of each sample. All values are expressed as the mean ± SE for n = 5–7 mice. * P<0.05, ** P<0.01 compared with a control and # *p*<0.05, ## *p*<0.01 compared with a HF.

The level of hepatic expression of fatty acid oxidation and lipogenesis-associated genes by exendin-4 treatment was assessed using real-time PCR (Figs. 3A–B). The expressions of adiponectin (*Adipoq*) and adiponectin receptor 2 (*Adipor2*) mRNA were decreased in the HF group compared with the control group and increased in the exendin-4- treated group compared with the HF group. A homologous gene of Adipor2, adiponectin receptor 1 (A*dipor1*), was also decreased in the HF group compared with the control group. However, no significant difference in the level of expression level of the A*dipor1* was observed between in the HF and exendin-4-treated groups. The HF group also exhibited a decrease in the expression of genes that are known to be regulated by peroxisome proliferator-activated receptor- α (*Ppara*), such as acyl-CoA oxidase (*Acox*) and medium chain acyl-coenzyme A dehydrogenase (*MCAD*) enzymes, which catalyze the key steps in peroxisomal and mitochondrial β-oxidation, respectively. On the other hand, the expressions of these genes were increased in the exendin-4-treated group compared with the HF group (Fig. 3A). As shown in Fig. 3B, sterol regulatory element binding protein-1c (*SREBP-1c*) and stearoyl CoA desaturase-1 (*Scd-1*) mRNA, which are key regulators of the *de novo* hepatic lipogenesis pathway, were decreased by exendin-4 treatment in HF- induced obese C57BL/6J mice. However, no significant difference was observed between the control and HF groups in the level of expression level of *SREBP-1c* mRNA. In addition, we observed changes in the expression of fatty acid synthase (*Fasn*) and acetyl-coenzyme A carboxylase alpha (*Acaca*) mRNA, which encode for lipogenic enzymes that are mediated by SREBP-1c. *Fasn* was decreased in the HF group compared with the control group and increased in the exendin-4-treated group compared with the HF group, but detected no change between the control and the exendin-4-treated group. Whereas, no significant difference in the level of expression level of the *Acaca* was observed between all groups. Therefore, these data suggest that the beneficial effects of exendin-4 on lipid metabolism are controlled through the regulation of fatty acid-oxidation rather than lipogenesis. Moreover, HF-decreased protein expression of phospho-Foxo1 and GLUT2, which are involved in hepatic glucose metabolism, was increased by exendin-4 treatment (Fig. 3C).

### Effect of exendin-4 treatment on liver fat accumulation

**Figure 4:**
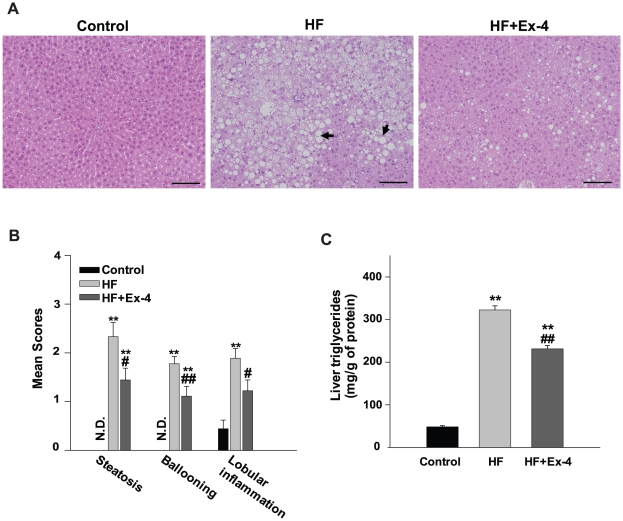
Histopathology of mice fed control (low fat), HF, HF+exendin-4 diet for 10 wks. Sections were stained with H&E. Arrows indicates lipid droplets. (A–C) Liver sections of mice fed control, HF and HF+Ex-4, respectively. (magnification, ×200). Scale bars = 500 µm. (D) NAFLD activity scores were evaluated semi-quantitatively: steatosis (0–3), lobular inflammation (0–2), and hepatocellular ballooning (0–2). N.D., not detected (E) Hepatic triglycerides were extracted from frozen tissue and measured by enzymatic assays. Values of TG were normalized to protein concentration. * *p*<0.05, ** *p*<0.01 compared with a control and # *p*<0.05, ## *p*<0.01 compared with a HF.

As shown in Fig. 4A–C, hepatic accumulation of lipids was significantly higher in the HF group compared with the control group and was decreased by exendin-4 treatment. In addition, mean scores for steatosis, ballooning and lobular inflammation based on H&E staining were significantly decreased in the exendin-4-treated group compared with the HF group (Fig. 4D). In the HF group, hepatic TG levels were dramatically increased compared with the control group, whereas it was decreased in the exendin-4 -treated group (Fig. 4E).

### Effect of exendin-4 treatment on PA-induced hepatic lipid accumulation *in vitro*

**Figure 5:**
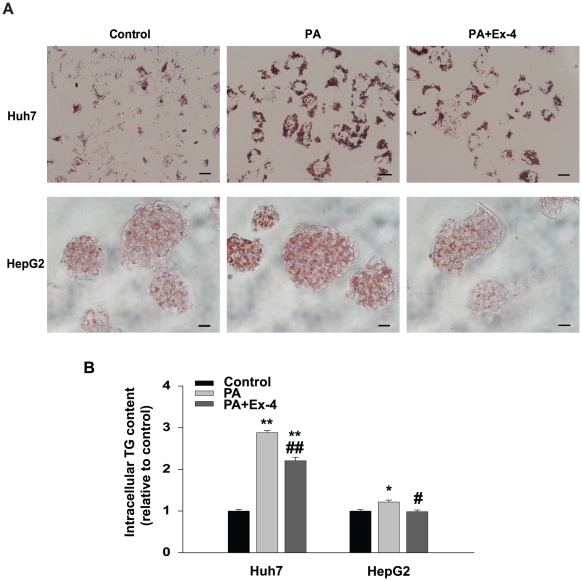
Effect of exendin-4 on PA-induced lipid accumulation in human hepatocytes. HepG2 and Huh7cells were treated with PA (0.4 mM) with or without Ex-4 (100 nM) for 24 h and lipid accumulation in cells was observed by Oil-red O staining. (A) HepG2 and Huh7 cells stained with Oil-red O were examined by light microscopy (magnification, ×400). Cells treated with 10% BSA, 0.4 mM PA and 0.4 mM PA+100 nM Ex-4; (B) intracellular lipid droplets were quantified using a spectrophotometer at 540 nm. * *p*<0.05, ** *p*<0.01 compared with a control and # *p*<0.05, ## *p*<0.01 compared with PA. Scale bars = 100 µm.

Lipid accumulation in HepG2 and Huh7 cells was examined by Oil-red O staining. Cells treated with PA, a saturated fatty acid, for 24 h had increased cellular lipid accumulation (Fig. 5). As shown by the photomicrographs, the significant changes of lipid droplets indicated that PA induced an increase of intracellular lipid content, and that exendin-4 could decrease PA-increased lipid accumulation. In addition, lipid content quantified using a spectrophotometer at 540 nm was consistent with visual staining analysis (Fig. 5B).

### Effects of exendin-4 treatment on regulation of GLP-1R, Sirt1 and AMPK *in vitro*

**Figure 6:**
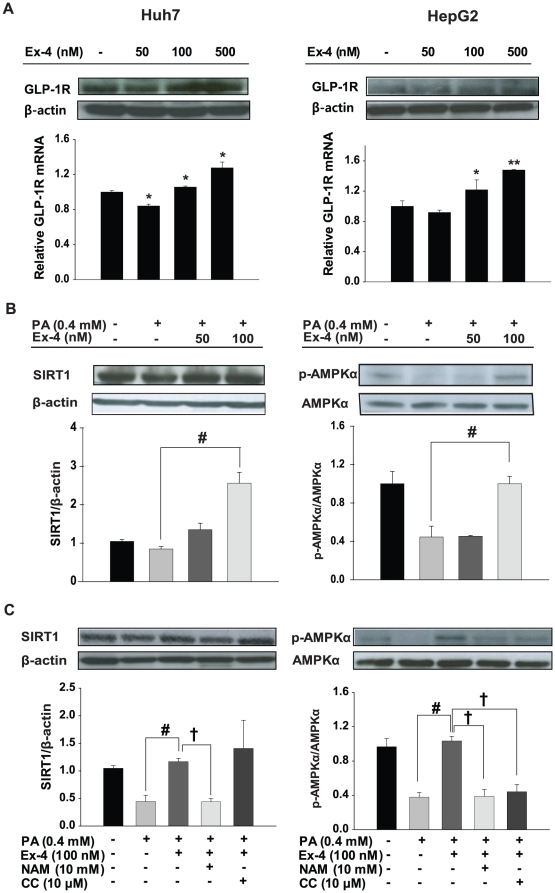
Regulation of GLP-1R, Sirt1 and AMPK by exendin-4 in HepG2 and Huh7 cells. (A) Cells were treated with 50 nM, 100 nM, or 500 nM Ex-4 for 24 h. GLP-1R and β-actin were measured by western blot and real time PCR. GLP-1R was normalized to β-actin. (B) Cells given 0.4 mM palmitic acid (PA) were treated with either vehicle or 50 nM to 100 nM exendin-4 for 24 h. (C) Cells given 0.4 mM palmitic acid were treated with 100 nM exendin-4 in the absence or presence of 10 mM nicotinamice (NAM) or 10 uM compound C (CC) for 24 h. (B–C) Sirt1, phosphorylated AMPKα at threonine 172, AMPK and β-actin were measured by western blot in HepG2 cells. Sirt1 and phosphorylated AMPKα were normalized to the β-actin and total AMPK of each sample, respectively. * *p*<0.05, ** *p*<0.01 compared with control, # *p*<0.05, ## *p*<0.01 compared with PA, and † *p*<0.05, †† *p*<0.01 compared with Ex-4.

To investigate whether the actions of exendin-4 are mediated by GLP-1R, HepG2 and Huh7 cells were treated with different doses of exendin-4. The expression of GLP-1R mRNA and protein was increased by 100 nM and 500 nM exendin-4 treatment, whereas it was decreased in cells treated with 50 nM exendin-4 compared with control cells (Fig. 6A).

Increased levels of Sirt1 and AMPK expression by exendin-4 were observed not only *in vivo*, but also *in vitro*. Exendin-4 (100 nM) treatment enhanced the protein expression of Sirt1 and phospho-AMPK in HepG2 cells treated with 0.4 mM palmitic acid (Fig. 6B). However, no effect on expression of sirt1 and phospho-AMPKα was observed in cells treated with 50 nM exendin-4, consistent with the results shown in Fig. 6A. Therefore, we decided to use a concentration of 100 -nM exendin-4 in further experiments.

Furthermore, to investigate whether AMPK is regulated by Sirt1, exendin-4 was added to HepG2 cells pretreated with or without either nicotinamide, a Sirt1 inhibitor, or Compound C, an AMPK inhibitor. As expected, in cells treated with PA, expression levels of Sirt1 and AMPK were decreased compared with cells treated with control and exendin-4. Also, nicotinamide significantly repressed Sirt1 and AMPKα expression compared with exendin-4 alone, whereas decreased Sirt1 expression by inhibition of AMPK in Compound C treatment was not observed. These data suggest that Sirt1 is an upstream regulator of AMPK in hepatocytes (Fig. 6C).

## Discussion

In this study, exendin-4 treatment resulted in a reduction of body weight and improvement in fat accumulation in liver tissues of a HF-induced obese mouse model. Together, we demonstrated that exendin-4 administration affected hepatic lipid metabolism via the Sirt1 signaling cascade, which are involved in fatty acid oxidation and lipid and glucose metabolism. However, interestingly the effects of exendin-4 treatment on appetite failed to coincide with those of previous studies, which reported a reduction in both body weight and appetite by exendin-4 administration [16]. This finding, that exendin-4 limited HF-induced weight gain despite no change in food intake (figure 1), is thought to be due to markedly increased energy expenditure, which was correlated with increased fatty acid oxidation. Recent studies showed that weight loss could decrease hepatic steatosis and insulin sensitivity [17] and a direct effect of GLP-1 on hepatocytes of human subjects with NASH could be mediated by activating of genes involved in fatty acid oxidation and insulin sensitivity [18]. In other words, it suggests that the effect of exendin-4 on liver is associated with weight loss.

GLP-1R is known to be widely expressed in pancreatic islets, lung, brain, heart, kidney, and stomach, and recently reported studies have focused mainly on the role of exendin-4 treatment in pancreatic islets. In addition, recent studies have demonstrated that GLP-1R is also present in human and rat hepatocytes [8], [9], and hepatic GLP-1 R expression is decreased in human subjects with NASH [18]. Furthermore, our results revealed that HF treatment decreased expression of GLP-1R in mouse hepatocytes, and that the expression of GLP-1R was increased by exendin-4 treatment in a dose-dependent manner in human hepatocytes, suggesting that GLP-1 might act directly on liver signaling.

Sirt1 and AMPK are signaling molecules that control hepatic lipid metabolism by deacetylation of acetylated lysine residues on histones and various transcriptional regulators, depending on intracellular NAD^+^/NAD ratios and phosphorylation via an increase in the AMP/ATP ratio, respectively [19], [20]. In the present study, we showed that Nampt expression was induced by exendin-4 treatment. The Nampt/visfatin enzyme catalyzes the reconversion of NAM to NAD^+^ and is required for Sirt1 activity [15]. Therefore, it is suggested that exendin-4 treatment increases Sirt1 expression via the NAD biosynthesis pathway.

Recently, an association was demonstrated between Sirt1 and AMPK signaling. Specifically, Sirt1 was found to regulate AMPK activity via modulation of Lkb1, a major upstream kinase of AMPK in hepatic cells in animal models [21]–[23]. On the other hand, AMPK enhances SIRT1 activity by increasing cellular NAD+ levels in mouse skeletal muscle [24]. Evidence for a Sirt1/AMPK signaling mechanism by exendin-4 treatment was observed in this study. In our *in vitro* study, the Sirt1 inhibitor nicotinamide decreased AMPKα expression, whereas a decrease in Sirt1 expression by an AMPK inhibitor, compound C, was not observed. These results indicate that Sirt1 is an upstream factor of AMPK, at least in hepatocytes.

AMPK activated by phosphorylation plays an important role via phosphorylation of its downstream genes in β-oxidation of fatty acid and glucose uptake [25]. Fatty acid oxidation in liver by 5-aminoimidazole-4-carboxamide ribonucleoside (AICAR) and metformin administration, an oral hypoglycemic agent, are also mediated by the activation of AMPK [26], [27]. In our study, HF significantly decreased the expression of genes associated with fatty acid oxidation, including the *Adipoq* and *Ppara* target genes, such as *MACD* and *Acox*, which are key enzymes involved in mitochondrial and peroxisomal β-oxidation, respectively. Adiponectin, as the key regulator of fatty acid oxidation in skeletal muscle and liver, increases AMPK and PPARα ligand activities. At this time, adiponectin-mediated modulation of AMPK depends on LKB [25], [26]. These actions of adiponectin are mediated by Adipor1 and Adipor2, which serve as receptors for globular and full-length adiponectin. Indeed, it has been reported that Adipor1 and Adipor2 are abundantly expressed in skeletal muscle and liver, respectively [28]. In our experiments, expression of Adipor2 was increased in the exendin-4-treated group compared with HF group, whereas no change in expression of Adipor1 was observed between the exendin-4-treated group and HF group. Thus, this result suggests that the antidiabetic effects of exendin-4-increased adiponectin are mediated by Adipor2. Further, these results may clarify whether one of the hepatic functions of exendin-4 treatment is through modulation of the fatty acid oxidation-associated signal transduction pathway.

In this study, histological images of HF mice showed a significant increase of lipid droplets in liver tissues. Further, exendin-4 treatment inhibited the development of NAFLD in HF-induced hepatic steatosis and plasma TG levels in HF-induced obese C57BL/6J mice. A previous study reported that exendin-4 impairs hepatocyte *de novo* lipogenesis by decreasing mRNA expression for *SCD-1*, *SREBP-1c*, and *ACC* in ob/ob mice [9]. For this reason, we expected that extendin-4 would regulate not only fatty acid oxidation, but also lipid metabolism, for the treatment of NAFLD. However, *Fasn*, which is lipogenic enzyme mediated by SREBP-1c, was decreased in the HF group, and change of *Acaca* was not observed between all groups. Higher level of lipogenic enzyme in the control group than the HF group can be explained by the difference in carbohydrate contents of the respective diets, due to the fact that dietary carbohydrate are transformed to fat via *de novo* lipogenesis processes.

In addition, conflicting results between previous studies and the present study with respect to expression of lipogenesis-associated genes may be due to the use of different mouse models, namely genetically and diet-induced obese mice. The results presented here showing that the effects of exendin-4 for improvement of hepatic steatosis give weight to a stimulatory role of exendin-4 on fatty acid oxidation rather than lipogenesis. In this study, animals were studied in the fasted state. Thus, it can be difficult to conclude about the changes in gene expression observed for genes encoding enzymes involved in lipogenesis. It could be better to perform experiments in feeding state.

The prevalence of NAFLD is associated with impaired glucose metabolism [29], whereby control of hepatic gluconeogenesis and glucose uptake is essential for maintenance of normal blood glucose concentrations. In this study, HF reduced hepatic phopho-Foxo1 and GLUT2 expression. FOXO1 and TORC2, which have been reported to promote gluconeogenesis, are repressed by SIRT1, resulting in decreased hepatic glucose production and improved glucose tolerance [30], [31]. In agreement with our data, exendin-4 improves glucose metabolism by enhancing hepatic insulin signaling and glucose uptake in the diabetic state [8], [32], [33]

Although exenatide does not function as a direct insulin sensitizer, there are reports on the positive effects of GLP-1 on NAFLD in human [10], [34]. A clinical trial using exenatide to assess drug safety in diabetics over an average period of 3.5 years revealed that patients with liver injury as assessed by elevated liver enzymes at baseline showed significant improvement in liver enzymes [34]. However, these effects were not reported to be associated with weight reduction. Another case report noted significant improvement in hepatic steatosis measured by liver spectroscopy after 44 weeks of treatment with exenatide [10]. Although the results from animal studies cannot be directly applied to humans due to the genetic differences across the species, the effects of exenatide on hepatic steatosis in humans is one of the interesting and promising aspects of incretin that has to be clarified through well-designed clinical trials in humans.

In summary, this study reveals that expression of hepatic GLP-1R is decreased by HF administration in mice, whereas it is directly proportional to exendin-4 concentration in human hepatocytes. In HF-treated mice, exendin-4 treatment ameliorated inflammation and steatosis, and increased sirt1 by exendin-4 lead to activation of AMPK and its downstream target genes, involving fatty acid oxidation. Therefore, the present study suggests that exendin-4 treatment may attenuate fat accumulation and improve glucose metabolism in liver tissues through the activation of Sirt1 signaling cascade, and further may serve as a therapeutic agent for fatty liver disease associated with metabolic diseases, such as obesity and T2DM.
